# Noise Is Not Error: Detecting Parametric Heterogeneity Between Epidemiologic Time Series

**DOI:** 10.3389/fmicb.2018.01529

**Published:** 2018-07-12

**Authors:** Ethan O. Romero-Severson, Ruy M. Ribeiro, Mario Castro

**Affiliations:** ^1^Theoretical Biology and Biophysics Group, Los Alamos National Laboratory, Los Alamos, NM, United States; ^2^Laboratorio de Biomatematica, Faculdade de Medicina, Universidade de Lisboa, Lisbon, Portugal; ^3^Grupo Interdisciplinar de Sistemas Complejos and DNL, Universidad Pontificia Comillas, Madrid, Spain; ^4^Department of Applied Mathematics, School of Mathematics, University of Leeds, Leeds, United Kingdom

**Keywords:** stochastic, deterministic, epidemiology, panel data, random effects, fixed effects

## Abstract

Mathematical models play a central role in epidemiology. For example, models unify heterogeneous data into a single framework, suggest experimental designs, and generate hypotheses. Traditional methods based on deterministic assumptions, such as ordinary differential equations (ODE), have been successful in those scenarios. However, noise caused by random variations rather than true differences is an intrinsic feature of the cellular/molecular/social world. Time series data from patients (in the case of clinical science) or number of infections (in the case of epidemics) can vary due to both intrinsic differences or incidental fluctuations. The use of traditional fitting methods for ODEs applied to noisy problems implies that deviation from some trend can only be due to error or parametric heterogeneity, that is noise can be wrongly classified as parametric heterogeneity. This leads to unstable predictions and potentially misguided policies or research programs. In this paper, we quantify the ability of ODEs under different hypotheses (fixed or random effects) to capture individual differences in the underlying data. We explore a simple (exactly solvable) example displaying an initial exponential growth by comparing state-of-the-art stochastic fitting and traditional least squares approximations. We also provide a potential approach for determining the limitations and risks of traditional fitting methodologies. Finally, we discuss the implications of our results for the interpretation of data from the 2014-2015 Ebola epidemic in Africa.

## 1. Introduction

Mathematical models play an increasingly central role in the analysis of infectious disease data at both the within-host and epidemiological levels (Perelson et al., 1996; Heesterbeek, 2000; Molina-París and Lythe, 2011). The traditional modeling approach involves formulating a set of structural assumptions about the processes involved, such as infection, recovery, death, etc. Often, these structural assumptions are then implemented in terms of differential equations, predominantly ordinary (ODE), but sometimes partial (PDE), or delayed (dODE) differential equations. The advantage of this approach is its amenability for both analytical treatment and powerful numerical and fitting algorithms even for non-linear problems. We will refer to those approaches collectively as *deterministic*.

However, stochasticity is an intrinsic feature of infections at multiple levels from the cellular/molecular world to the level of epidemics (Süel et al., 2006; Bressloff and Newby, 2013). The deterministic framework conceptualizes all deviation from the model prediction as **error**. For example, in a simple univariate linear regression we say that the data are equal to a linear predictor plus some error. Put another way, we can say that error is the density of the data conditional on the model. However, stochasticity generates intrinsic fluctuations in the underlying dynamics of a system (for instance, in the number of secondary cases an incident case generates), even when the process follows the structural model envisaged. That is, stochasticity generates **noise**, which we define as the set of outcomes that are consistent with a fixed set of assumptions (i.e., a model).

One of the central challenges of using the deterministic framework is to delineate its limitations (Roberts et al., 2015). If the world and its data truly are stochastic, then how much of a problem is it to conflate noise with error? Likewise, how much information in the data are we neglecting by treating all deviation as uninformative error? To what extent is the assumption of deterministic dynamics plus error providing misleading results?

This question is not gratuitous as some parameters estimated within the deterministic framework, such as the basic reproduction number (*R*_0_), are often invoked to quantify the aggressiveness of a pathogen and to determine the conditions under which a pathogen will go extinct (Dietz, 1993; Heffernan et al., 2005) or to create public health information such as risk maps (Hartemink et al., 2011).

The potential problems in applying the deterministic framework can become even more pronounced when we have data that represent multiple realizations of a heterogeneous stochastic process. For example, a set of viral load profiles in different infected individuals (e.g., primary HIV infection; Ribeiro et al., 2010) or epidemic curves in different regions (e.g., cases of Ebola in multiple counties of the same country; Krauer et al., 2016), that is, any data that can be represented as a panel over discrete units. In those scenarios, an important question is whether the variability seen between units can be attributed to a genuine difference in the process that generated the data (e.g., some parameters of the dynamics are different for each unit), simple stochastic fluctuation, or a mixture of the two, in addition to measurement error. Given a common error model across the units, the deterministic framework assumes that all deviation that cannot be explained by error must be due to parametric variability between units, that is the units are fundamentally different from one another. For this reason, the deterministic framework is ill-suited to tackle the question of stochastic effects.

We address in this paper two related questions regarding modeling of panel data: (i) can we use a stochastic modeling approach to partition variability into stochastic and parametric components? and (ii) can we quantify the bias induced by modeling the data by a deterministic approach with error? Put in other words, is there a best and a good-enough fitting method for the practitioner? In section 2.1, we consider two simple structural models that will help us emphasize the essence of the problem without having to invoke unnecessary complexities that may cloud our main arguments. In section 3.1, we present our approach to analyze those models, which will then be used to benchmark comparisons between traditional (deterministic) fitting methods and more sophisticated stochastic ones, that we explore in section 3.2. As a case study, in section 4, we compare deterministic and stochastic modeling approaches to data from the 2014-2015 Ebola epidemic in West Africa. We use epidemic data from multiple counties of those countries that were most heavily affected. If one thinks of each county as a realization of some epidemic generating process, then the relevant question is whether differences between the counties can be accounted for by stochastic variability or if it is possible to detect a signal for different growth rates of the epidemic in different counties. Finally, in section 5 we summarize our results and discuss the implications of our work.

## 2. Methods

### 2.1. Simulated data

The general framework we employ is to simulate data *in silico* from two structural models, birth-only or birth-death process (see Karlin, 2014), by a discrete-time stochastic simulation and then fit those data using both deterministic and stochastic methods under a variety of assumptions.

The code used to generate the data and fit the models is given in Appendix A. We simulate panel data according to the following process 
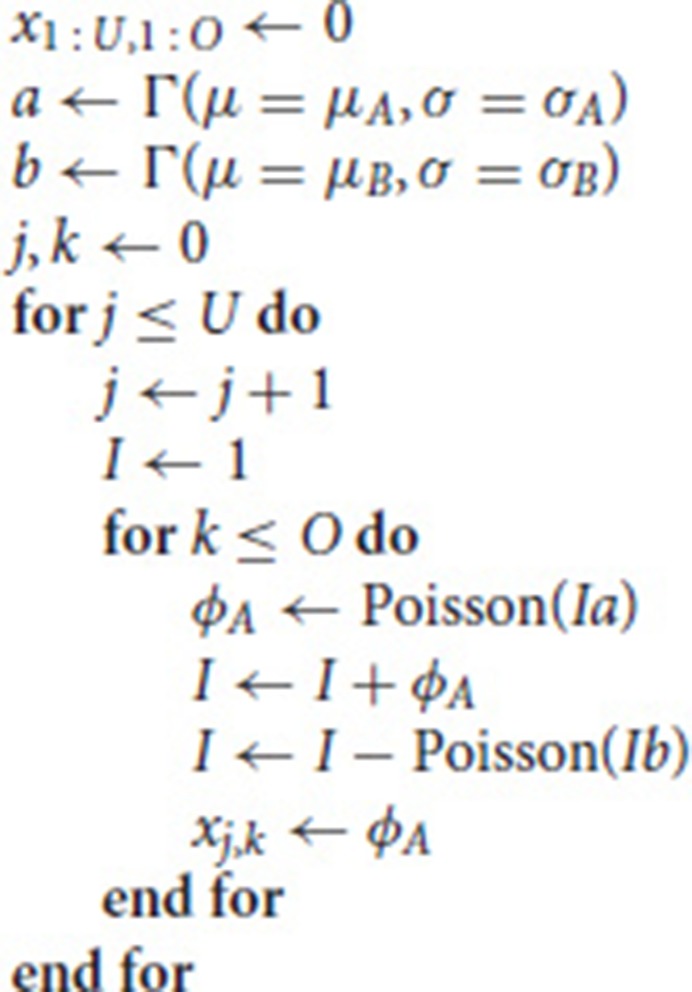
 where *U* is the number of units in the panel, *O* is the number of observations (time points) per unit, and *x*_*j,k*_ is the number of new infected cases in each time period *k* for unit *j*—this is the output of the simulation used for the fits described below. If the number of deaths exceeds the infected population size, *I*, this variable is set to 0. These simple models capture both the initial exponential growth phase when infected population sizes are small and stochastic die out that is common in many epidemiological processes. For simplicity, we focus only on the early stages of the epidemic, i.e., the approximately exponential phase in the growth of infected individuals. Note that throughout we use arbitrary time units.

Each simulated data set is specified by 6 parameters: mean growth rate, μ_*A*_; standard deviation of the growth rate, σ_*A*_; mean death rate, μ_*B*_; standard deviation of the death rate, σ_*B*_; the number of units in the panel, *U*; and the number of observations per unit, *O*. From this we consider 4 possible scenarios: birth-only without parametric variability (μ_*B*_ = σ_*B*_ = σ_*A*_ = 0), birth-only with parametric variability (μ_*B*_ = σ_*B*_ = 0), birth-death without parametric variability (σ_*A*_ = σ_*B*_ = 0), and birth-death with parametric variability. In all cases with parametric variability, we assume a Gamma distribution for the respective parameter (where μ and σ are the corresponding mean and standard deviation). We chose the Gamma distribution because it can easily be re-parameterized into its mean and standard deviation, which makes interpreting the parameters straightforward.

We set up four sets of simulated experiments to explore the effects of (1) model misspecification, (2) the number of observations per unit, (3) the number of units in the panel, and (4) the heterogeneity in parameters (growth rates) between units (see Table 1 for reference).

**Table 1:** Summary of groups of numerical experiments, the aim of each experiment and the figure summarizing the main results for each case.

**Experiment #**	**Description**	**Model**	**Results**
1	Effect of model misspecification	Birth only and birth-death	**Figure 3**
2	Effect of number of observations	Birth only	**Figure 4**
3	Effect of number of units in the panel	Birth only	**Figure 5**
4	Effect of parametric variance	Birth only	**Figure 6**

*In all cases (in particular in Experiment 4), we compare fitted parameters using the stochastic and deterministic methods described in section 3.1. In all cases, we made use of simulated data with and without random effects to account for the impact of parametric variance*.

In the first set of experiments, we simulate data from a birth-only process without parametric variability (μ_*A*_ = 0.15), birth-only with parametric variability (μ_*A*_ = 0.15, σ_*A*_ = 0.02), birth-death without parametric variability (μ_*A*_ = 0.25, μ_*B*_ = 0.1), and birth-death with parametric variability (μ_*A*_ = 0.25, σ_*A*_ = 0.02, μ_*B*_ = 0.1, σ_*B*_ = 0.01). In each case, we assume (*U* =) 20 units per panel and (*O* =) 20 observations per unit, at equal time intervals. We then fit each of these four data sets using each of four possible models (birth or birth-death with and without random effects) with both stochastic and deterministic approaches for a total of 32 fits.

In the next three sets of experiments we use the birth-only model with parametric variability and the default parameters μ_*A*_ = 0.15, σ_*A*_ = 0.02, *U* = 20, *O* = 20. In the second set of experiments, we vary the number of observations per unit (*O* ∈ {10, 20, 30, 40, 50}), in the third set of experiments we vary the number of units in the panel (*U* ∈ {10, 20, 30, 40, 50}), and in the fourth set of experiments we vary the heterogeneity in growth rates (σ_*A*_ ∈ {0.01, 0.02, 0.03, 0.04, 0.05}).

### 2.2. Parameter inference

To infer the parameter values, we use a fitting scheme based on simulations that can account both for the intrinsic stochasticity of the process and the potential variation among individuals. Here all model formulations (both stochastic and deterministic versions) are fit using the iterated filtering method implemented in the R library pomp (King et al., 2016). This approach allows us to fit all the models to the data using the same framework and likelihood functions, such that the model fits are all comparable. We specifically use the iterated filtering for panel data (IFPD) formulation detailed in Romero-Severson et al. (2015). Code used to specify the pomp process are given in Appendix A.

Models were fit using 5,000 or 15,000 particles for the deterministic and stochastic models respectively. For stochastic fits, the density of the number of incident cases in the *kth* time period of the *jth* unit, *x*_*j,k*_, is assumed to be Poisson(*x*_*j,k*_|*I*_*j,k*−1_α) were *I*_*j,k*_ is the simulated number of extant infected cases in the *kth* time period of the *jth* unit and α is the growth rate, which itself may be sampled from a Gamma distribution. For the deterministic fits, *x*_*j,k*_ is simply *x*_*j,k*_ = α*I*_*j,k*−1_.

To obtain confidence intervals (CIs) for the parameters, we used a profile likelihood method (Romero-Severson et al., 2014) where the parameter of interest was varied over a grid of values and the likelihood was calculated, by refitting the data allowing all other parameters to be free. We used the mif2 method (King et al., 2016) in pomp. A local regression (loess) curve was fitted to the profile likelihood curve and both the MLE and CIs were calculated from the interpolated curve (King et al., 2015, 2016).

### 2.3. Ebola data and analysis

The Ebola case count data was compiled from publicly available datasets published by the World Health Organization (from the “Ebola Data and Statistics” section of the WHO website). Case counts were stratified by country and county of origin. All descriptive analyses were done on the full data. However, to fit the models to the data using the simulation-based method described, we restrcited the data in the following way.

(i) For every county, we define time = 1 as the first week where the total number of cases is larger or equal to 1.
(ii) We truncated the data at 10 weeks after that time, in order to have homogeneous sets (same number of points) during the approximately exponential initial growth of the epidemic. To emphasize this latter point, we re-plot the data in linear-log scale.
(iii) Finally, we removed those counties where the data does not include at least 10 data points.

Note that in the simulated data, we assumed no measurement error in time or in number of infected. However, this is not a good assumption for real epidemiological data. Thus, for the Ebola data, we fit a modified version of both the deterministic and stochastic birth-only model accounting for measurement error (e.g., missed cases and reporting delays) in a simple way, by assuming that the number of new cases is distributed according to a Negative Binomial, rather than a Poisson, conditional on the simulated state of the system at the previous time. We re-parameterize the typical NB(*n, p*) as $\text{NB}\left(\delta ,\frac{\mu }{\mu +\delta }\right)$ where μ is the mean of new cases and δ is an overdispersion parameter such that $\underset{\delta \to \infty }{\lim}\text{NB}\left(\delta ,\frac{\mu }{\mu +\delta }\right)=\text{Poisson}\left(\mu \right)$. Therefore, the mass of the data conditional on the simulated state of the system is $\text{NB}\left(y_{j,k}|\delta ,\frac{x_{j-1,k}a}{x_{j-1,k}a+\delta }\right)$. The parameter δ controls the level of overdispersion (smaller values, more overdispersion) in the data conditional on the simulated state and is free (estimated) for each point in the likelihood profiles. This formulation puts the stochastic and deterministic models on a level playing field in that the deterministic model can model variance between epidemic trajectories with increased overdispersion rather than increased population-level heterogeneity. The deterministic and stochastic models were fit with 5,000 and 15,000 particles, respectively, for each value in the profiles (**Figure 10**).

## 3. Results

### 3.1. Motivation: noise as parametric heterogeneity

Traditional inference is based on maximum likelihood estimates of some well-defined functions. For instance, for the cases considered here (pure birth and birth-death) an ODE-based deterministic approximation provides differential equations that, upon solving, can be fit to the data to determine the parameters (μ_*A*_ = α and μ_*B*_ = γ) that best describe the data (see Table 2, and Appendix B for a succinct derivation for the pure birth case). Similarly, the stochastic version of those models can be solved and in that case one could also fit the mean and variance of a given observable (last two rows in Table 2), and indeed higher moments.

**Table 2:** Number of infected, new cases and total cases for the birth and the birth-death processes as defined in the deterministic (top part of the table) and stochastic (bottom part) approaches.

	**Birth process**	**Birth-death process**
Differential equation	$\frac{dI}{dt}=\alpha I$	$\frac{dI}{dt}=\left(\alpha -\gamma \right)I$
Infected, *I*	*e*^α*t*^	*e*^(α − γ)*t*^
New cases per unit time, *N*	α*e*^α*t*^	α*e*^(α−γ)*t*^
New cases in Δ*t*, *N*_*t*_	*e*^α*t*^(*e*^αΔ*t*^ − 1)	$\frac{\alpha }{\alpha -\gamma }e^{\left(\alpha -\gamma \right)t}\left(e^{\left(\alpha -\gamma \right)\Delta t}-1\right)$
Total cases, *T*	*e*^α*t*^	$\frac{\gamma }{\alpha -\gamma }\left(\frac{\alpha }{\gamma }e^{\left(\alpha -\gamma \right)t}-1\right)$
Mean of infected, 〈*I*〉	*e*^α*t*^	*e*^(α−γ)*t*^
Variance of infected, $\sigma_{I}^{2}$	*e*^α*t*^(*e*^α*t*^ − 1)	$\frac{\alpha^{2}-\gamma^{2}}{{\left(\alpha -\gamma \right)}^{2}}e^{\left(\alpha -\gamma \right)t}\left(e^{\left(\alpha -\gamma \right)t}-1\right)$

*In all cases, the epidemic starts with one infected case, namely, I(0) = 1. Here we only consider models without parametric variability (σ_A_ = σ_B_ = 0)*.

In these cases, as the models are linear, both deterministic and stochastic predictions for the average are the same (because averaging and integrating the evolution equation are exchangeable operations). However, the latter has the benefit that it also allows to fit the variance of the data (thus, in principle, increasing the reliability of the inferred parameters).

**Figure 1:**
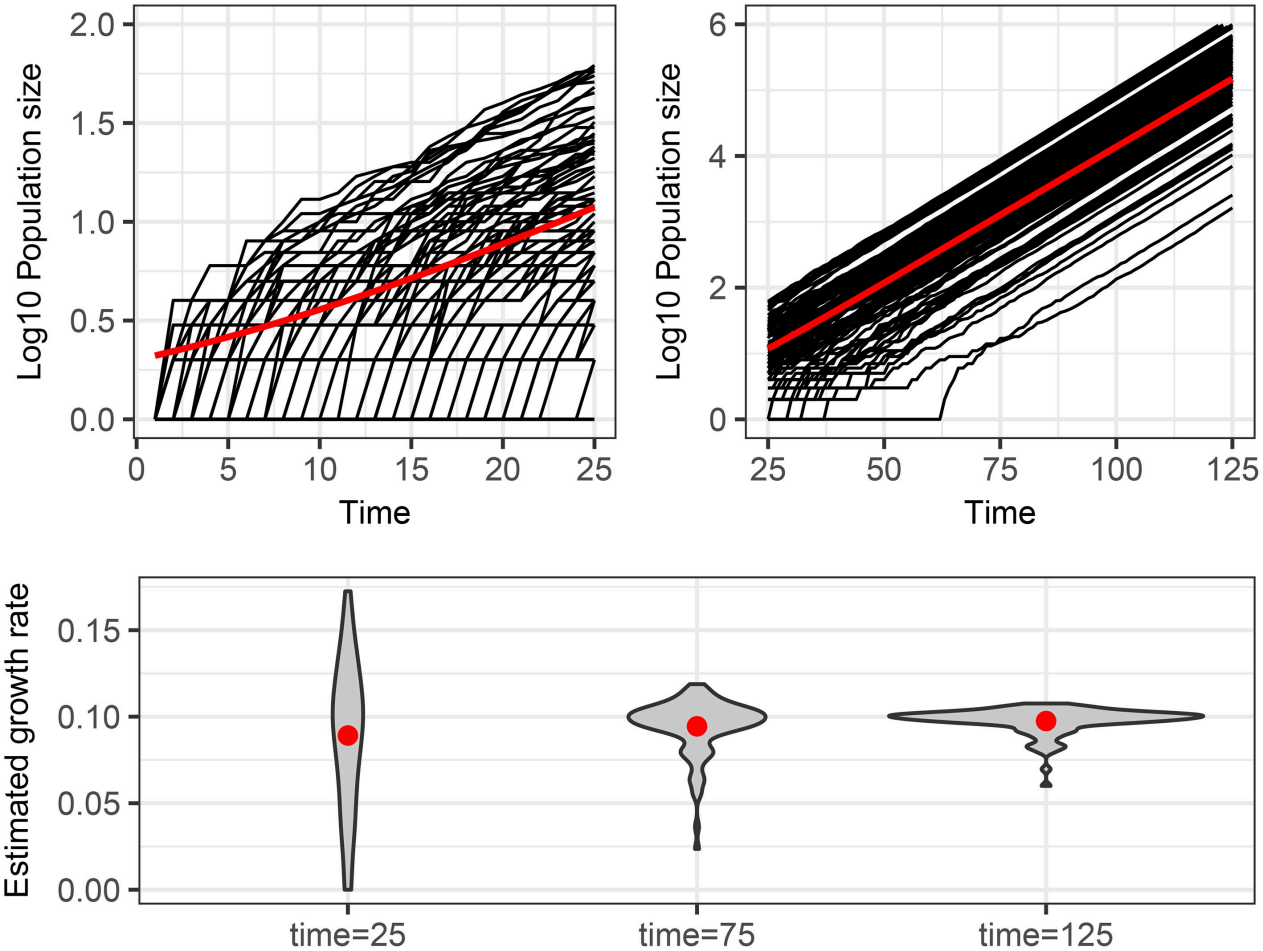
Stochastic realizations of a pure birth process and distributions of deterministic estimation of the growth rate at different times. Top figures show 100 trajectories from a continuous time pure-birthh process with parameter α = 0.1 over two time scales. The only difference between each trajectory is intrinsic stochastic variability. The red line shows the expected population size assuming a deterministic process, which is also the mean number of infected of the stochastic process if there is no parametric variability. The bottom plot shows the distribution of estimated growth rates obtained by fitting a linear model to the log_10_ of the population size for each of the 100 trajectories from time 0 to times 25, 75, and 125. The red dots indicate the mean of the estimated growth rates, which are all close to the true value of 0.1.

The main point that we wish to address is how to interpret different trajectories of an intrinsic stochastic process. To illustrate this point, Figure 1 shows 100 realizations of the simple stochastic pure birth model with rate parameter α = 0.1 time-unit^−1^ measured without error at integer times. If we use a naive deterministic approach (top of Table 2), we fit *I*(*t*) = *e*^α*t*^ to each trajectory (data set) and estimate α independently, obtaining a distribution for this parameter (Figure 1, bottom panel). If this process were observed at time 25, it would be tempting to conclude that there is a high degree of heterogeneity in the growth rates of these epidemics. Even by time 75, when the expected population size is over 1,000, we still see a large heterogeneity in the estimated rates.

If we used the stochastic version of the pure birth process (bottom of Table 2), by definition we would assume that there was just one value for the α parameter and could fit the mean and variance (and possibly other moments) of the trajectories to estimate that growth rate.

Another possible deterministic fitting approach is to allow for random effects, where we assume an underlying distribution (e.g., normal) for the growth rate parameter (α) and allow each trajectory to be the realization of a pure birth process with parameter drawn from that distribution (Gelman and Hill, 2007). In this case, the estimation method yields the parameters of the distribution (i.e., the mean and variance). This is a mixed effects approach, where we still assume no stochasticity and that all differences are due to parametric variability.

This approach of assuming parametric variability can also be used with the stochastic version of the model. In fact, it is instructive to analyze in more detail such situation by calculating analytically the distribution of the number of infected accounting both for the stochasticity of the process and the parameter distribution for the pure birth process.

If we assume that the growth rate, α, is distributed according to a normal, $\alpha \sim N\left(\mu_{A},\sigma_{A}\right)$, then the probability of having *I*(*t*) total infected is the product of the geometric distribution for fixed α, which is the solution of the pure birth process, (see Allen, 2010), and the normal distribution for α, namely

$$\begin{array}{l} P\left(I|\mu_{A},\sigma_{A},t\right)=P\left(I|\alpha ,t\right)P\left(\alpha |\mu_{A},\sigma_{A}\right)\\ \;=\left[p{\left(1-p\right)}^{I-1}\right]\frac{e^{-{\left(\alpha -\mu_{A}\right)}^{2}/2\sigma_{A}^{2}}}{\sqrt{2\pi \sigma_{A}^{2}}} \end{array}$$

where *p* = *e*^−α*t*^. Therefore,

(1) $$\begin{array}{r} P\left(I|\mu_{A},\sigma_{A},t\right)=\frac{{\left(1-e^{-\alpha t}\right)}^{I-1}e^{-\frac{{\left(\alpha -\mu_{A}\right)}^{2}}{2\sigma_{A}^{2}}-\alpha t}}{\sqrt{2\pi \sigma_{A}^{2}}},\;I=1,2,\ldots \end{array}$$

From this expression, we can obtain the mean and variance of *I*, including the contributions of both stochasticity and parametric variability (see also Appendix C)

(2) $$\begin{array}{r} \langle I\rangle =e^{\mu_{A}t+\frac{\sigma_{A}^{2}t^{2}}{2}} \end{array}$$

and

(3) $$\begin{array}{r} \sigma_{I}^{2}=e^{\mu_{A}t+\frac{\sigma_{A}^{2}t^{2}}{2}}\left(e^{\mu_{A}t+\frac{\sigma_{A}^{2}t^{2}}{2}}\left(2e^{\sigma_{A}^{2}t^{2}}-1\right)-1\right) \end{array}$$

(These expressions reduce to the forms in Table 2, when σ_*A*_ = 0). It is worth noting that both the mean and the variance of *I* depend on μ_*A*_ and σ_*A*_, suggesting that an ODE or stochastic fit to the mean ignoring parametric variability would estimate the growth rate incorrectly.

These four different ways to fit the same data set (e.g., Figure 1) beg the question of which one is the best approach and whether that depends on the data containing actual parametric variability or not. On the other hand, the explicit knowledge of the stochastic form of σ_*I*_, both in the presence of parametric variability (expression 3) and pure stochastic variability (Table 2), suggests the definition of a quantity, *R*^2^ (analogous to a coefficient of determination) as

(4) $$\begin{array}{r} R^{2}=\frac{\sigma_{\text{param}}^{2}}{\sigma_{\text{param}}^{2}+\sigma_{\text{noise}}^{2}}=1-\frac{\sigma_{\text{noise}}^{2}}{\sigma_{I}^{2}} \end{array}$$

For the pure birth process (see Appendix C for details):

(5) $$\begin{array}{r} R^{2}=\frac{\frac{1}{2}\sigma_{A}^{2}t^{2}e^{\mu_{A}t}\left(6e^{\mu_{A}t}-1\right)}{e^{\mu_{A}t}\left(e^{\mu_{A}t}-1\right)+\frac{1}{2}\sigma_{A}^{2}t^{2}e^{\mu_{A}t}\left(6e^{\mu_{A}t}-1\right)}\left(≃\frac{3\sigma_{A}^{2}t^{2}}{1+3\sigma_{A}^{2}t^{2}}\right) \end{array}$$

**Figure 2:**
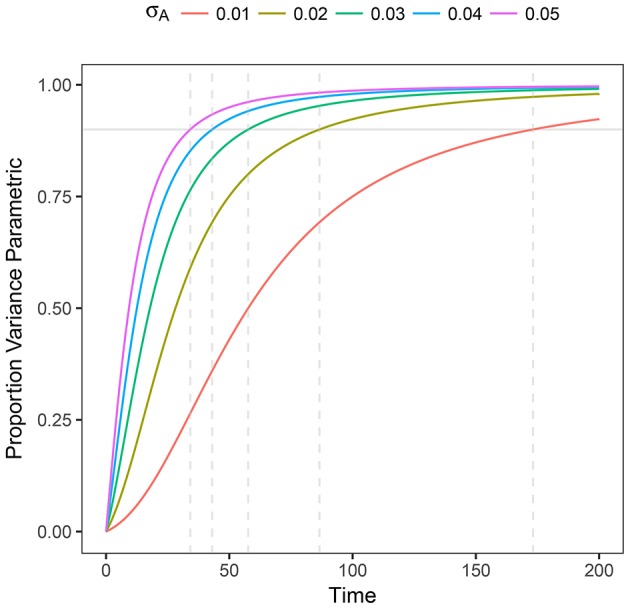
Plot of *R*^2^ as a function of time for heterogeneous stochastic exponential growth. Each line shows *R*^2^ for the specified level of σ_*A*_ assuming μ_*A*_ = 0.1. The horizontal gray line indicates 90% of the variance being due to parametric heterogeneity; the dashed vertical gray lines indicate the time at which each line reaches 90%.

This expression helps us to determine (in a prescriptive way) whether the process is governed by stochasticity (*R*^2^ → 0) or by parametric variability (*R*^2^ → 1). Also, as it can be expected, the variance at shorter times is governed by pure random fluctuations but as time proceeds, parametric variance, if present, is increasingly more relevant. We plot *R*^2^ as a function of time in Figure 2

To analyze these issues in more detail, we now use *in silico* generated data fitted in multiple ways, with and without stochastic effects and with and without assuming parametric variability, to assess the quality of the parameter estimation.

### 3.2. Comparison of fitting methods with simulated data

In Appendix D (Tables I to IV) we summarize the fitted parameters discussed in the Sections 3.2.1 to 3.2.4.

#### 3.2.1. Experiment 1: model misspecification

**Figure 3:**
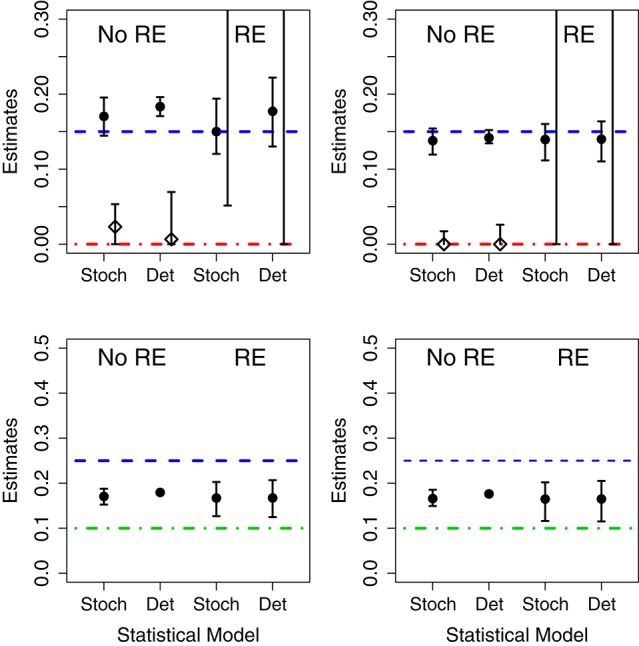
Results of fits when using mismatched structural models. The symbols correspond to the estimates of the growth rate (circles) and death rates (diamonds) under different scenarios. In the **(Left)**, the data was generated without parametric variability and in the **(Right)** the data was generated with parametric variability. Top row: results of fits with a birth-death model to data generated by a pure birth process. In each case, we used stochastic or deterministic fits, without (“No RE”) or with (“RE”) random effects. The horizontal dashed blue lines indicate the value μ_*A*_ (birth rate) and the dot-dashed red line the value of σ_*A*_ used in the data generation. Bottom row: results of fits with a pure birth model to data generated by a birth-death process. The horizontal dashed blue lines stand for μ_*A*_ and the dot-dashed green lines for μ_*B*_. In all cases, the vertical whiskers are the 95% CI obtained in the fits. Note that the estimates of the death rate for the random effects fits (in the top panel) are off the plot, and only the bottom segments of the whiskers are visible).

We fit 4 models (birth-only and birth-death, with and without random effects) using both deterministic and stochastic model formulations allowing us to consider the effect of both model structure misspecification and other model assumptions. Parameter estimates for each data set are given in Table I in Appendix. Also, in Figure 3 we summarize succinctly the main conclusions of this section.

##### 3.2.1.1. Correct model

When the data are generated without population heterogeneity (i.e., σ_*A*_ = σ_*B*_ = 0) and fit with the correct structural model, both the deterministic and the stochastic fits have reasonable point estimates and their confidence intervals (CIs) contain the true parameter value (shown in Table I in Appendix). However, CIs on the death rates are very broad suggesting that the incidence data are only weakly informative. When we introduce population heterogeneity into both the data and fits, the stochastic fit still contains the true parameter values in its CIs; although fitting all 4 parameters leads to very broad estimates for the mean and standard deviation of the death rate. The deterministic model, however, is unable to estimate either the mean or standard deviation of the growth rates correctly.

##### 3.2.1.2. Random effects in the model but not the data

When the fit attempts to estimate random effects when no parametric variability is actually present, the CIs for the estimated standard deviation of the parameters in the stochastic fits contain 0, while the deterministic CIs do not. That is, the deterministic model finds evidence for population-level heterogeneity when none actually exists.

##### 3.2.1.3. Random effects in the data but not the model

When there is population-level heterogeneity in the data but the model assumes that there is none, the stochastic fit still obtains correct point estimates and CIs of the mean effects for both the birth-only and birth-death models. However, in the deterministic fits the CIs for the mean effects did not contain the true values of the growth rates.

##### 3.2.1.4. Death in the data but not in the model

When fitting the birth-death data with a birth-only model, we found that, in both the stochastic and deterministic fits, the estimate of the growth rate is close to the net growth rate (i.e., birth rate minus death rate). However, if we allow random effects on the growth rate, the deterministic fits finds a very high level of heterogeneity in the growth rate when none actually exists. The CI for the standard deviation of the parameter in the stochastic fit correctly contains 0, suggesting limited evidence for heterogeneity in growth rates.

##### 3.2.1.5. Death in the model but not in the data

Conversely, if there is death in the model, but not in the data, both the fixed effects stochastic and deterministic fits the CIs for the death rate correctly contained 0. However the deterministic fit overestimated the growth rate while the stochastic fit did not.

#### 3.2.2. Experiment 2: number of units in the panel

**Figure 4:**
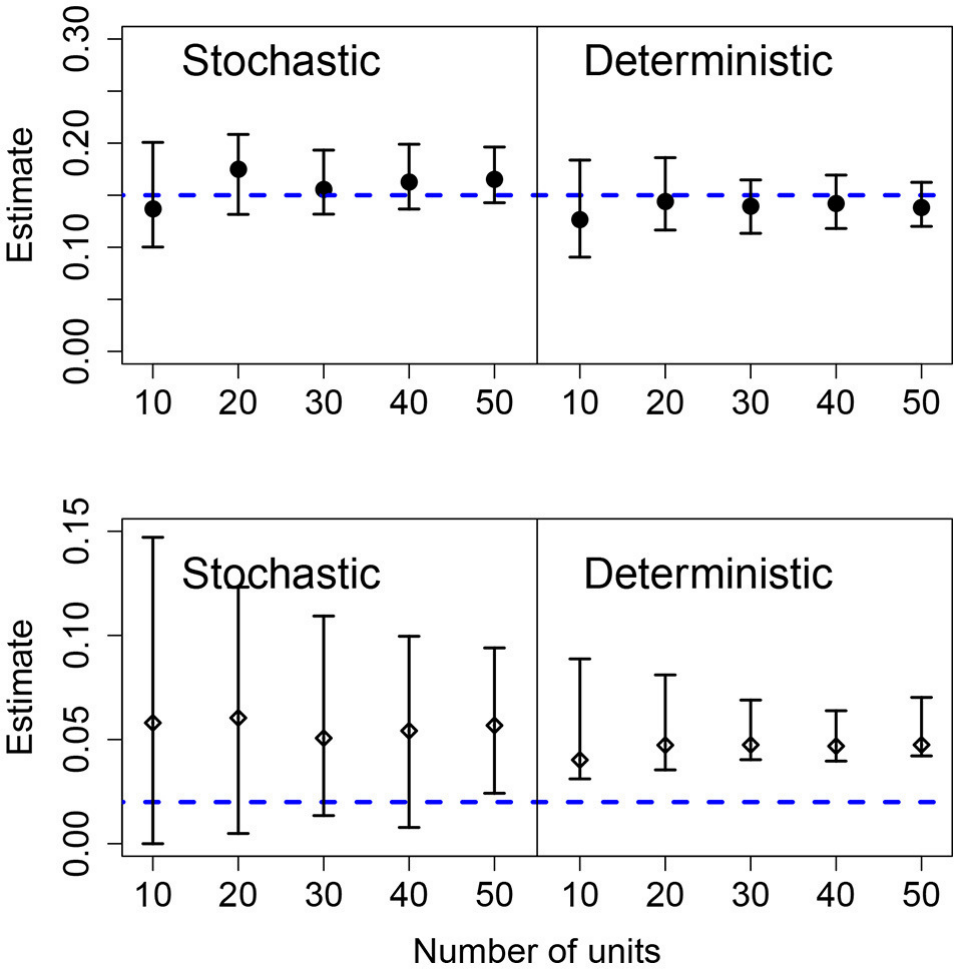
Results of fits when there is a variable number of units. The data in all cases was generated by a pure birth process with parametric variability and fit with a birth-only model. The top row shows the estimates for the mean growth rate with stochastic or deterministic fits, and the bottom row the estimate of the standard deviation of the growth rate. The horizontal dashed blue lines indicate the parameter values used in the data generation. The vertical whiskers are the 95% CI obtained in the fits. In each case, the number of observations per unit was *O* = 20, the growth rate was α = 0.15 and the standard deviation of the growth rate was σ = 0.02.

Results for data generated by a pure birth process, with different number of units in the panel, are shown in Figure 4. Using the stochastic or the deterministic fits resulted in point estimates for the mean growth rates that were very close to the mean value and the CIs contain the true value for all cases. Increasing the number of units in the panel causes slightly narrower CIs for the mean growth rate as well. The standard deviation of the growth rates was correctly estimated in the stochastic model for all but one case; however, the deterministic model overestimated the population-level heterogeneity in all cases. Also, as the number of units in the panel increases, the CIs narrow suggesting a higher degree of certainty in an incorrect conclusion.

#### 3.2.3. Experiment 3: number of observations per unit

**Figure 5:**
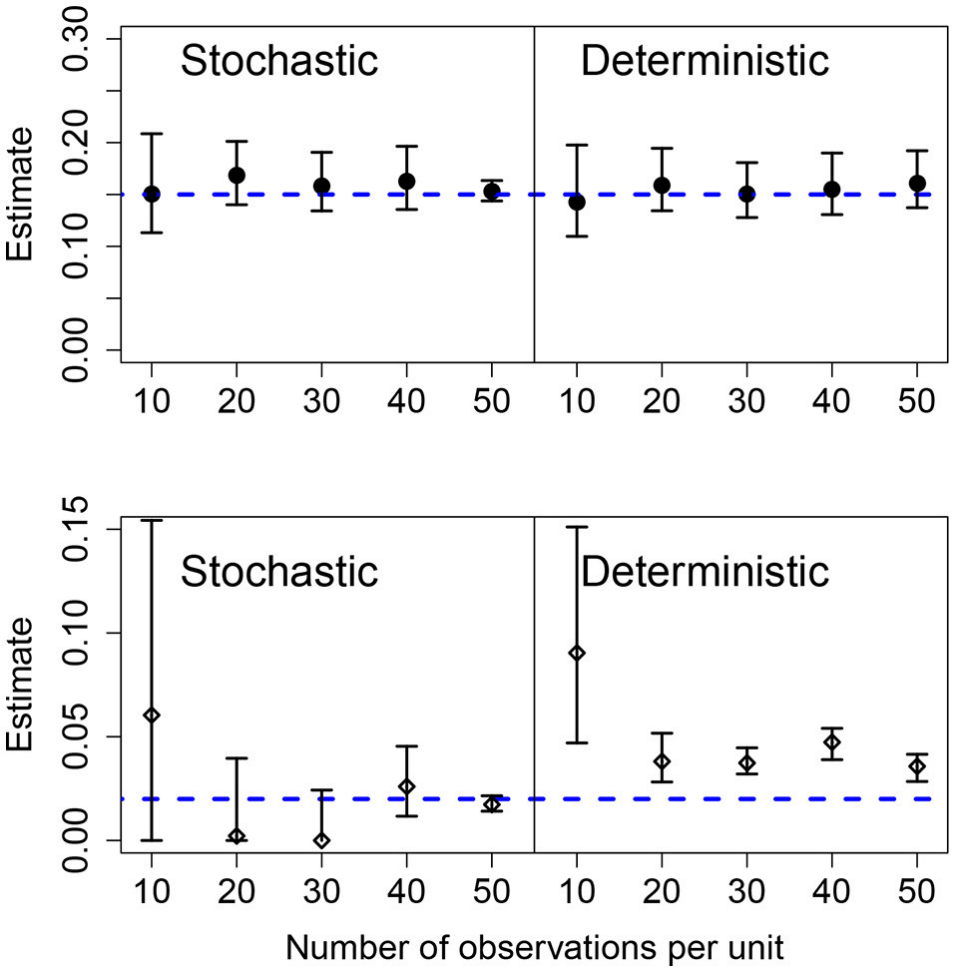
Results of fits when there is a variable number of observations in each unit. The data in all cases was generated by a pure birth process with parametric variability. The top row shows the estimates for the mean growth rate with stochastic or deterministic fits, and the bottom row the estimate of the standard deviation of the growth rate. The horizontal dashed blue lines indicate the parameter values used in the data generation. The vertical whiskers are the 95% CI obtained in the fits. In each case, the number of units was *U* = 20, the growth rate was α = 0.15 and the standard deviation of the growth rate was σ = 0.02.

The effects of increasing the number of observations per units was similar to increasing the number of units in the panel. For both the stochastic and deterministic fits, the mean growth rates where correctly estimated. As before, the deterministic fit consistently overestimated the standard deviation in the growth rates and increasing the number of observations per unit led to narrower but wrong CIs. Increasing the number of observations per unit is more efficient at improving the accuracy of the estimation compared to increasing the number of units in the panel for the stochastic model. Results are shown in Figure 5.

#### 3.2.4. Experiment 4: increasing heterogeneity between units

**Figure 6:**
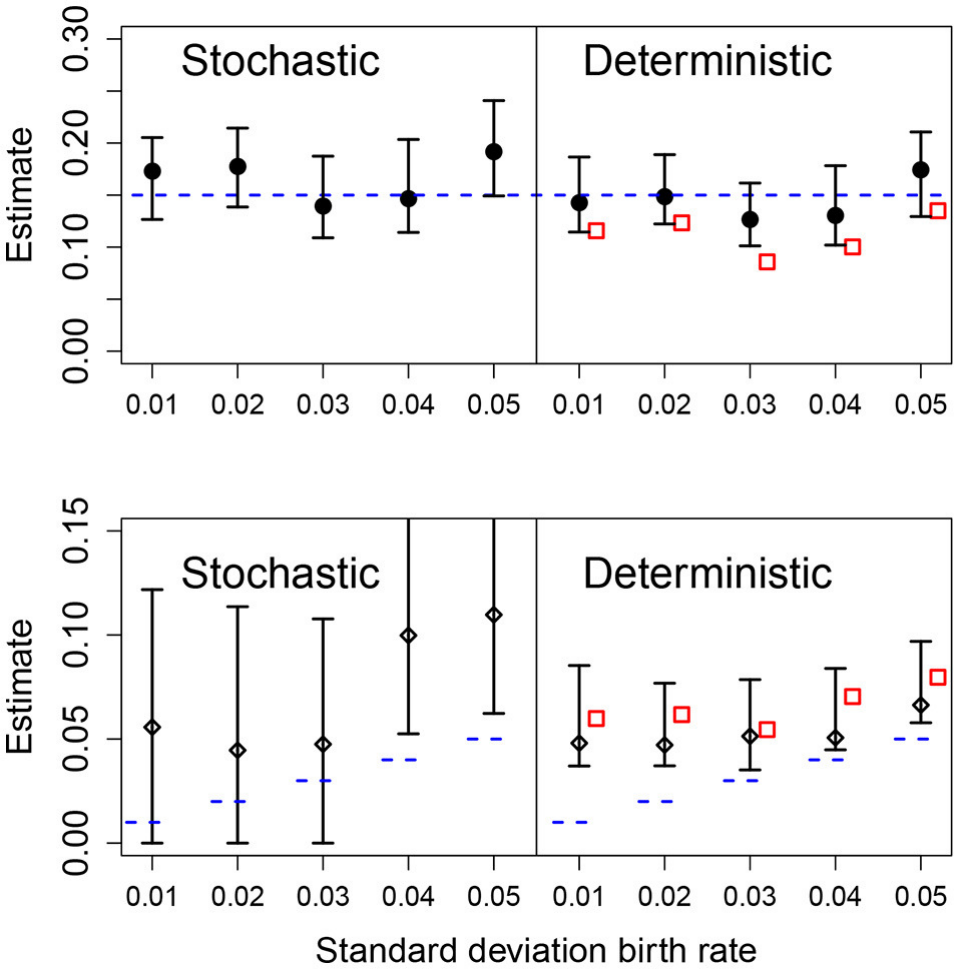
Results of fits with increasing standard deviation for the growth rate. The data in all cases was generated by a pure birth process with parametric variability. The top row shows the estimates for the mean growth rate with stochastic or deterministic fits, and the bottom row the estimate of the standard deviation of the growth rate. The horizontal dashed blue lines indicate the parameter values used in the data generation. The vertical whiskers are the 95% CI obtained in the fits. In the right panels, the red empty squares are the estimated values obtained from standard linear mixed-effect models (regression). In each case, the number of observations per unit was *O* = 20 and the numbers of units was *U* = 20.

We also analyzed the effect of different values for the heterogeneity of the parametric variability. As before, the deterministic fit consistently overestimated the level of heterogeneity regardless of the actual value of the standard deviation of the growth rate, however, these estimates became closer to the true value with increasing heterogeneity in the data. In the stochastic fits, when the heterogeneity was less than 0.04, the estimated CIs included the true parameter and increasing heterogeneity led to a narrower CI. At the highest heterogeneity levels the CI did not contain the true value; we found that using a stochastic fit to data with high levels of parametric heterogeneity leads to numerical instability making estimation of the CIs difficult. Results are shown in Figure 6.

### 3.3. Quantifying parametric variability with *R*^2^

**Figure 7:**
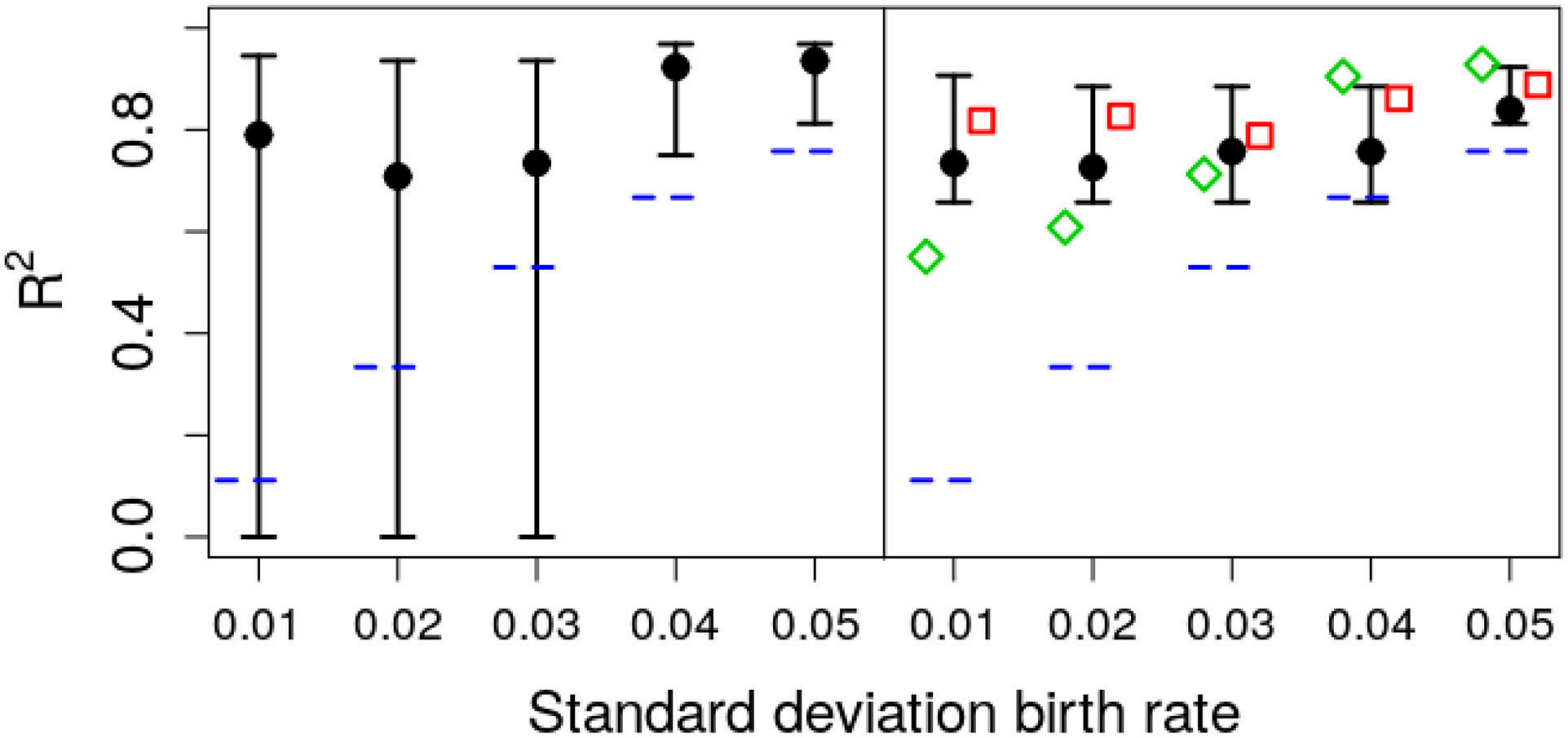
Estimated *R*^2^ with increasing standard deviation for the growth rate. The data in all cases was generated by a pure birth process with parametric variability. The horizontal short dashed lines indicate the parameter values used in the data generation. The left panel corresponds to the stochastic fits and the right panel to the deterministic fits, where the vertical whiskers are the 95% CI obtained in the fits. The red empty squares in the right panel stand for the value of *R*^2^ calculated with Equation (5) at time *t* = 20 with parameters estimated using standard linear mixed-effect models (regression). The empty green diamonds are an alternative way to estimate *R*^2^ using the empirical data variance and the theoretical (stochastic) noise variance, Equation (8). In each case, the number of observations per unit was *O* = 20.

As shown in Figure 6, the deterministic CIs do not include the real value of σ_*A*_, albeit the estimate of μ_*A*_ is accurate enough. To test the ability of different methods to quantify the relevance of parametric variance vs. noise (through *R*^2^), we use the estimation of σ_*A*_ from the different methods with Equation (5), at the final observation, *t* = 20. The results are shown in Figure 7. Note that the stochastic prediction, at least, is able to include the real *R*^2^ inside the whisker, especially at low values of parametric variability. This means that this fitting method is able to capture (in a probabilistic way) the cases where parametric variance is not as relevant as fluctuations.

We have used throughout simulation-based inference, because it allows us to compare directly likelihood profiles between stochastic and deterministic implementations of the models. Nevertheless, it is worth remembering that traditional methods (based, loosely speaking, on regression) are usually the preferred way to estimate parameters from the data. This is not a matter of taste but of computational efficiency. Even for the simple models in the present work, simulation-based inference is computationally expensive (and, as such, not suitable as of writing for models with many parameters). Thus, for the sake of completeness we discuss briefly the role of regression-based methods in our framework and fit the data in Experiment 4 using a standard linear mixed-effect model (Gelman and Hill, 2007). We find that this fit results in a systematic underestimation of the mean, μ_*A*_ (red squares in Figure 6 top), and in an overestimation of the standard deviation σ_*A*_ (red squares in Figure 6 bottom).

While Equation (5) was derived under the assumption of an unerlying stochastic process, and traditional methods ignore the stochasticity of the underlying process, we can still use hybrid information to obtain a *rough* estimate the relative weight between noise and parametric variance. We can mix both approaches (linear mixed-effect models and stochatic predictions) in two ways: In the first one (corresponding to the red empty squares in Figure 7) we use μ_*A*_ and σ_*A*_ from the linear mixed-effects model fit to the data in Equation (5). The second method, consists in calculating the empirical variance of the data and the expected value of the noise variance from Equation (8) and calculate *R*^2^ using Equation (4). Remarkably, inspection of Figure 7 (green empty diamonds) suggest that using this second method, the estimated value of *R*^2^ is sometimes closer to the original one.

In summary, combining standard methods with analytical results coming from the exact solution of the stochastic process might be useful to estimate the level of noise in the data. Notwithstanding, in all cases, this hybrid method used to calculate *R*^2^ also overestimates the true value.

## 4. Case study: the 2014-15 Ebola epidemic

### 4.1. Heterogeneity of epidemic spread of Ebola

**Figure 8:**
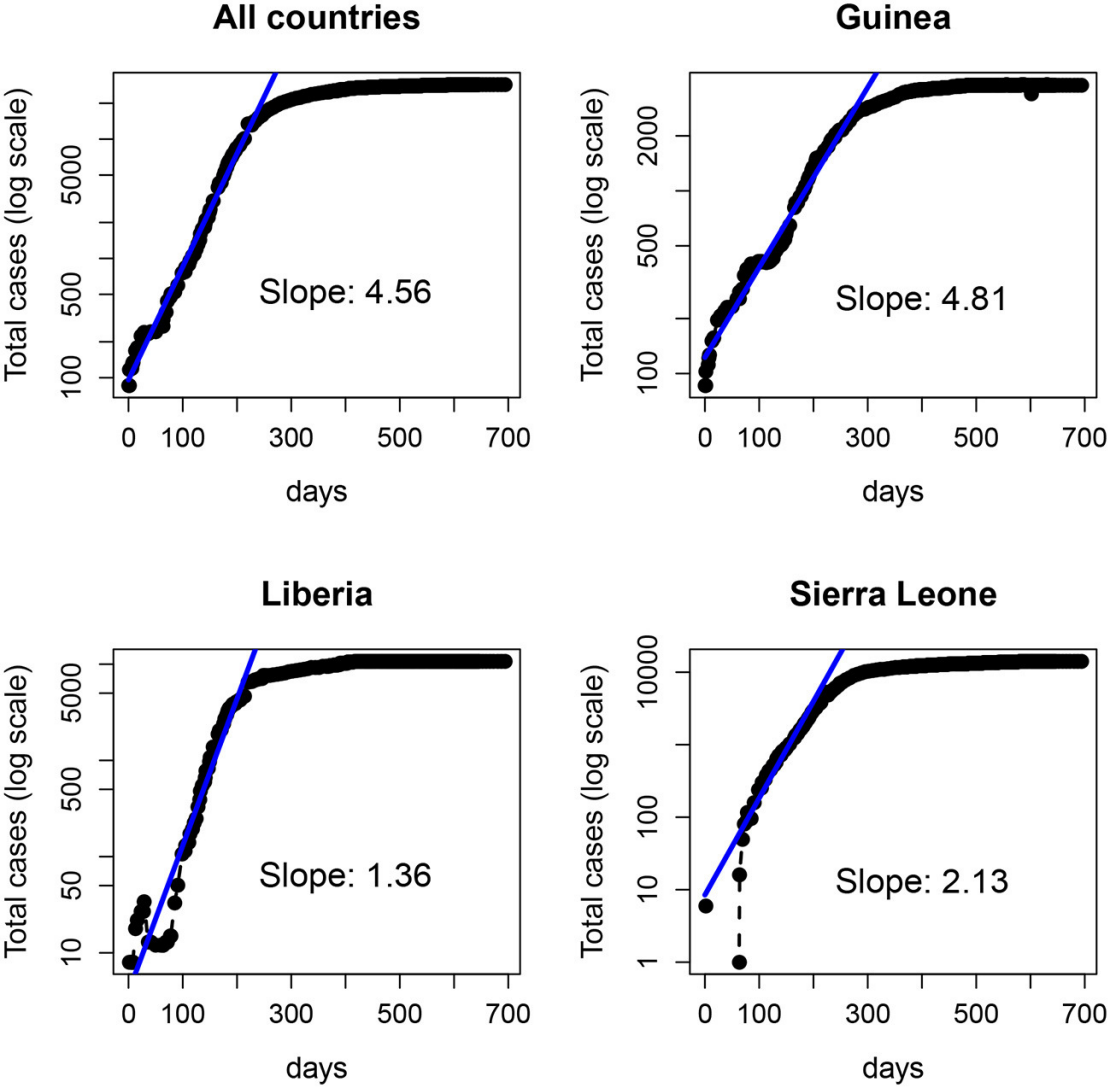
Number of Ebola cases (logarithmic scale) of the 2014-15 Ebola epidemic. **(Top left)** total number of cases in the three countries: Guinea **(Top right)**, Liberia **(Bottom left)**, and Sierra Leone **(Bottom right)**. The solid line represents the fit of an exponential function to the data in each panel over the first 200 days (~29 weeks).

In Figure 8 we show the total number of cases reported for the 2014-15 Ebola epidemic in Guinea, Liberia and Sierra Leone. In each case, the solid line is the fit of an exponential function to the data for the first 29 weeks. Despite the fluctuations (specially in the first days) the fit provides an (apparently) accurate account for the growth during those early weeks. Note that the estimated slopes are highly variable among countries. Since for simple models, the slope in the exponential fit (α) is proportional to the basic reproductive number minus one (*R*_0_ − 1) (Heffernan et al., 2005), with this approach one would conclude that the severity of Ebola in different countries is highly variable. Indeed, this variability has been reported for the 2014-15 epidemic (with *R*_0_ ranging between 1.51 and 2.53), see (Althaus, 2014; Kucharski et al., 2015; Krauer et al., 2016), as well as for earlier outbreaks (Chowell et al., 2004).

**Figure 9:**
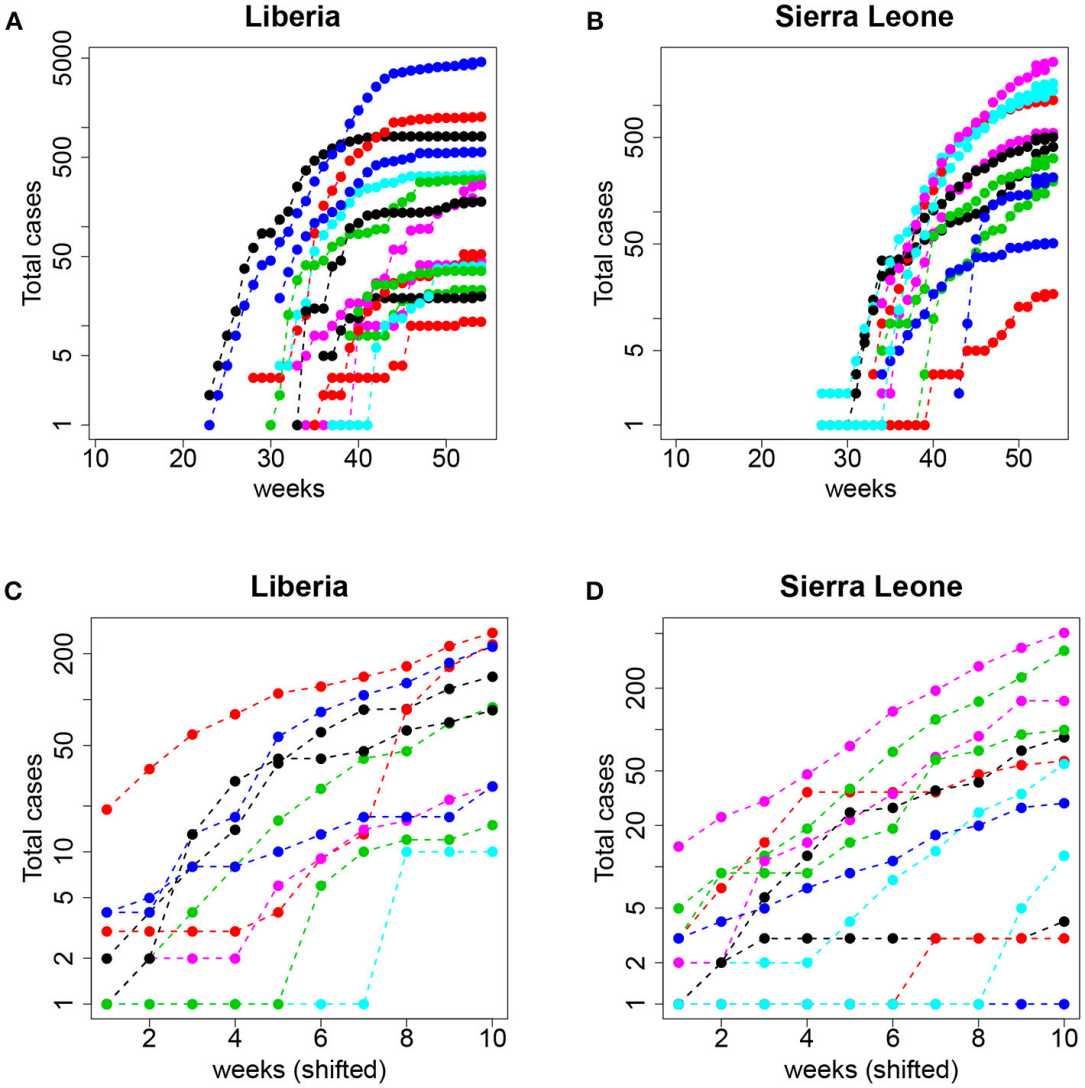
Top: Total number of cases (logarithmic scale) per county in **(A)** Liberia and **(B)** Sierra Leone. Bottom: The same but aligning week 1 to the date of the first event with *I*≥1 and restricting to the first 10 observations (see text for details); **(C)** Liberia and **(D)** Sierra Leone.

From a traditional deterministic approach we might come to two conclusions: (1) The Ebola epidemic is well described by a deterministic model that predicts accurately the initial exponential growth and (2) the epidemic was more aggressive in Guinea, followed by Sierra Leone and Liberia. However, a closer inspection of the data (collected by counties) before the aggregation shows a different picture. In Figure 9, we plot the same dataset (for Liberia and Sierra Leone) but separately for the different counties.

Now, the conclusions that can be drawn are more nuanced and perhaps contrary to the picture of uniform growth suggested by Figure 8. On the one hand, the starting dates of the epidemic in different counties are highly variable, and the initial slopes (the plot is in logarithmic scale) also display a large variability. This suggests that assigning a simple value per country (and, consequently a single *R*_0_) can be misleading and lead to erroneous interpretations and, more importantly, interventions or policies. On the other hand, and this is what we are interested in, this fine grained view of the data begs for a stochastic approach to fitting. Even when the data is aggregated (which tends to smooth the underlying stochasticity), the initial part of the curves are reminiscent of the trajectories in Figure 1 (left panel).

### 4.2. Ebola model fits

**Figure 10:**
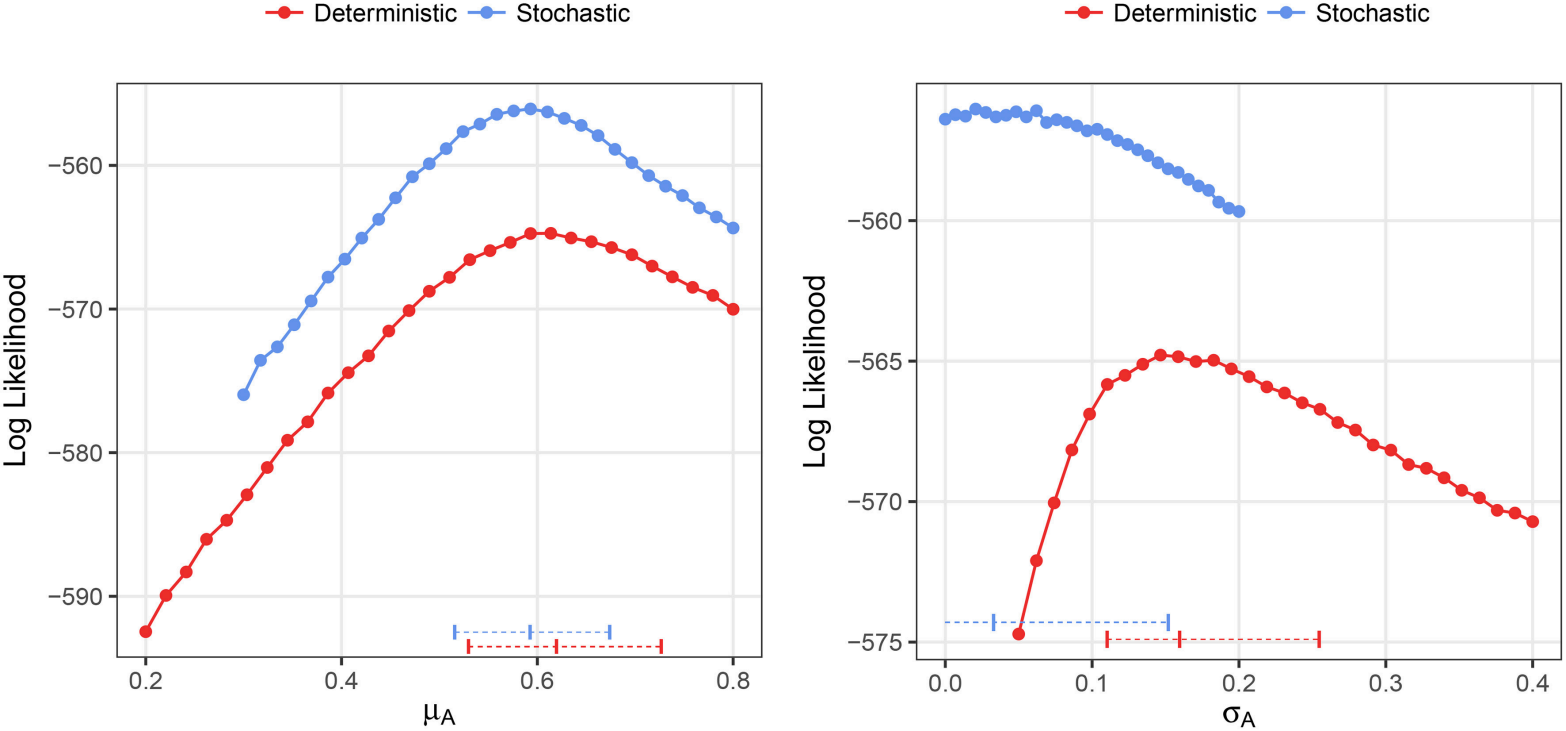
Profile likelihood plots for the parameter estimates for the Ebola data. The plot on the left shows the profile likelihood for the mean growth rate, μ_*A*_, while the plot on the right shows the profile likelihood for the standard deviation of growth rates over counties, σ_*A*_. Horizontal dashed lines indicate the MLE and 95% CIs for the parameter estimates. The overdispersion parameter was free to vary in the calculation of each point along the profile.

We fitted both deterministic and stochastic versions of a birth-only model with random effects to the Ebola data, allowing for negative binomial measurement error (see section 2.3 for details). The stochastic model was, in terms of the likelihood values, objectively better than the deterministic model (−556.4 vs. −565.0) despite being identical in all respects except stochasticity. The estimate of the mean growth rate was nearly identical in both models, 0.62, with CI (0.53, 0.73) deterministic and 0.59, with CI (0.52, 0.67) stochastic (Figure 10). However, the deterministic model found a very high level of heterogeneity, 0.16 CI (0.11, 0.25), while the stochastic model found low levels of heterogeneity, 0.03 CI(0, 0.15). In the stochastic model, the profile likelihood for the standard deviation in growth rates, σ_*A*_, suggests that the likelihood surface is virtually flat around very small values of σ_*A*_ (see Figure 10 right). However, in the deterministic model—even when we allow variable levels of overdispersion—the likelihood rapidly drops off as the heterogeneity decreases from the MLE.

Overall, these results show that, while deterministic fitting is as good as stochastic fitting to estimate the mean growth rates, it performs poorly as a predictor of the parametric variability. Specifically, using our definition of *R*^2^, and the MLE of σ_*A*_ = 0.03, obtained with the stochastic method, we can estimate the contribution of parametric variability to overall variability in the data. Using Equation (5) results in *R*^2^ ≃ 0.21. This analysis would suggest that, in the case of Ebola, 10 weeks after the start of the epidemic, around 79% of the measured variability could be attributed to noise rather than to inter-county differences. Taking into account that, as we showed in Figure 7, this empirical way to calculate *R*^2^ overestimates the true coefficient, the conclusion is even more substantiated. Doing the same calculation with the value obtained in the deterministic fitting, σ_*A*_ = 0.16, we get *R*^2^ ≃ 0.88, so we would conclude that 88% of the variability is due to true differences among counties.

## 5. Discussion and conclusions

The aim of modeling is not to capture every specific feature of the system under consideration but, rather, to describe succinctly the main mechanisms of the process and, ideally, to be able to differentiate among competing hypotheses (Ganusov, 2016). The art of modeling involves balancing multiple levels of complexity to achieve predictability, accuracy, and tractability. In this context, here we have added another concern: is the methodological approach suitable? Following an approach of keeping things simple, we have shown that even for the most basic cases, deterministic fitting methods, which assume that all variability is either error or parametric, provide misleading results. Although, not all aspects of the models were sensitive to the assumption of determinism, since for example the mean of a parameter was usually reasonably estimated.

This study is not a purely academic exercise on the role of fluctuations for small populations because our results point to important practical implications. A case in point is our example of the initial spread of the Ebola epidemic. Although different counties seem to have different growth rates, our fitting indicates that the variability is also well explained by stochastic (i.e., non-systematic) differences among the counties. This does not mean that there are no differences in epidemic spread among the counties, only that stochasticity alone is a statistically better and more parsimonious explanation. That is, when stochasticity is taken into account the evidence for differences in early growth rates is negligible.

The ability to accurately detect and measure heterogeneity is an important topic with practical implications. Take, for example, the expanding field of personalized medicine, where individual treatment plans may be designed under the potentially faulty assumption that there is heterogeneity in response to treatment regimes. Likewise, scientific resources may be wasted in a quest to search for individual-level correlates of heterogeneity that may not exist. Our results suggest that measuring heterogeneity in panel data time series is prone to bias and misinterpretation and that including more data in terms of additional observations per unit or increasing the number of units will not alleviate this bias caused by methodological misspecification.

In this regard, it is important to note that the simulation-based stochastic fits, generally speaking, appropriately partitioned variability into stochastic and parametric components even with relatively short time series. This means that such methods should be preferred for fitting data. However, there are practical issues with implementing stochastic fitting methods when the models are complex (e.g., multiple populations or many parameters) or the populations involved are large. This is because the computational resources needed and the time to fit a given model would be, in most cases, prohibitive. As an alternative, if a fully stochastic model is not possible, one could explore the possibility of using stochastic models for a limited time window (for instance, early on). Although, this will need the development of hybrid fitting methodologies. Generally, one should be cautious when interpreting the fit of deterministic models to panel data, since the observation of parametric heterogeneity or even structural heterogeneity in terms of model selection may be the result of overfitting stochastic fluctuation. Also, the term *R*^2^ can be estimated numerically for a given model to provide a warning of potential problems based on deterministic model fits.

In summary, here we analyzed the effect of neglecting stochastic noise (i.e., in addition to the error term) in panel data of biological time series. We found that deterministic approaches usually overestimate the parametric variability, although (at least in our simple models) the parameter average is less difficult to estimate. On the other hand, stochastic fitting, in general, did a good job of dividing variability between stochastic and parametric.

## Author contributions

All authors listed have made a substantial, direct and intellectual contribution to the work, and approved it for publication.

### Conflict of interest statement

The authors declare that the research was conducted in the absence of any commercial or financial relationships that could be construed as a potential conflict of interest.
