# Supplementary material for: Noise Is Not Error: Detecting Parametric Heterogeneity Between Epidemiologic Time Series
Source: Front Microbiol. 2018 Jul 12;9:1529. doi: 10.3389/fmicb.2018.01529 (PMC6052138; doi:10.3389/fmicb.2018.01529)
Supplement: Supplementary file 1 [file Presentation_1.pdf]

frontiers

Appendix to manuscript *Noise is not error: detecting parametric heterogeneity between epidemiologic time series*

Ethan Obie Romero-Severson [1], Ruy M. Ribeiro [1,2] and Mario Castro [3,4,*]

[1] *Los Alamos National Laboratory, Theoretical Biology and Biophysics Group, Los Alamos, New Mexico, USA.*
[2] *Universidade de Lisboa, Faculdade de Medicina, Laboratório de Biomatemática, Lisbon, Portugal*
[3] *Universidad Pontificia Comillas, Grupo Interdisciplinar de Sistemas Complejos (GISC) and DNL, Madrid, Spain.*
[4] *University of Leeds, School of Mathematics, Department of Applied Mathematics, Leeds, UK.*

Correspondence*:
Mario Castro
marioc@comillas.edu

APPENDIX

A. POMP CODE

To specify a `pomp` process we need to specify a `rprocess` function that simulates the state of the system conditional on a given parameter vector and a `dmeasure` function that returns the likelihood of the data given the simulated state at a given time. Inference was performed with `pomp` version 1.13.

The `rprocess` for the deterministic birth-death model is coded as

```
step.fn = Csnippet(
 "
␣␣double␣p1,␣p2,␣p3,␣p4,␣t1,␣t2,␣t3,␣t4;
␣␣t1␣=␣exp(mu_a);␣t2␣=␣exp(sig_a);␣t3␣=␣exp(mu_b);␣t4␣=␣exp(sig_b);
␣␣p1␣=␣pow(t1,2)/pow(t2,2);
␣␣p2␣=␣pow(t2,2)/t1;
␣␣p3␣=␣pow(t3,2)/pow(t4,2);
␣␣p4␣=␣pow(t4,2)/t3;
␣␣double␣a␣=␣rgamma(p1,␣p2);
␣␣double␣b␣=␣rgamma(p3,␣p4);
␣␣double␣curpop␣=␣1;
␣␣double␣S[100];
␣␣int␣i;
␣␣for(i=1;␣i<21;␣i++){
␣␣␣␣S[i]␣=␣curpop;
␣␣␣␣double␣nbirth␣=␣curpop␣*␣a;
```

```
    curpop = curpop + nbirth;
    double ndeath = curpop * b;
    curpop = curpop - ndeath;
    if(curpop<0)curpop=0;
  }
  S1=S[1];S2=S[2];S3=S[3];S4=S[4];S5=S[5];
  S6=S[6];S7=S[7];S8=S[8];S9=S[9];S10=S[10];
  S11=S[11];S12=S[12];S13=S[13];S14=S[14];S15=S[15];
  S16=S[16];S17=S[17];S18=S[18];S19=S[19];S20=S[20];S21=a;
  ")

rprocess = discrete.time.sim(step.fn)
```

The stochastic birth-death model is identical with the terms `curpop * a` and `curpop * b` replaced by `rpois(curpop * a)` and `rpois(curpop * b)` respectively. The `dmeasure` function is coded as

```
dmeasure = function(y, x, t, params, log, ...){
  a = x[21]; y = y[1:20]; x = x[1:20]
  pr = sum(dpois(y, x*a, log=T))
  if(log==F)pr = exp(pr)
  pr
}
```

The simulated data are generated with the following code

```
to.internal = function(mu, sig){
  p1 = mu^2/sig^2
  p2 = sig^2/mu
  c(p1,p2)
}

bd.sim = function(N, K, mu.A, sig.A, mu.B, sig.B){
  pa = to.internal(mu.A, sig.A)
  pb = to.internal(mu.B, sig.B)
  ret = data.frame(time=0:K)
  for(n in 1:N){
    if(sig.A<1e-50) a=mu.A else a = rgamma(1, pa[1], scale=pa[2])
    if(sig.B<1e-50) b=mu.B else b = rgamma(1, pb[1], scale=pb[2])
    sl = numeric(K+1)#state line
    dl = numeric(K+1)#data line
    sl[1] = 1; dl[1] = 1
    for(k in 2:(K+1)){
      n.birth = rpois(1, sl[k-1]*a)
      sl[k] = sl[k-1] + n.birth
      n.death = rpois(1, sl[k]*b)
      if(n.death > sl[k]) n.death = sl[k]
```

```
      sl[k] = sl[k] - n.death
      dl[k] = n.birth
    }
    ret[paste("d",n,sep="")] = dl
  }
  ret
}

A1 = list()
A1[[1]] = exp.sim(20, 20, 0.15, 0)
 early onA1[[2]] = exp.sim(20, 20, 0.15, 0.02)
A1[[3]] = bd.sim(20, 20, 0.25, 0, 0.1, 0)
A1[[4]] = bd.sim(20, 20, 0.25, 0.02, 0.1, 0.01)
saveRDS(A1, "simdat1.rda")

A2 = list()
for(i in seq(10,50,10)){
  A2[[length(A2)+1]] = bd.sim(20,i,0.15,0.02,0,0)
}
saveRDS(A2, "simdat2.rda")

A3 = list()
for(i in seq(10,50,10)){
  A3[[length(A3)+1]] = bd.sim(i,20,0.15,0.02,0,0)
}
saveRDS(A3, "simdat3.rda")

A4 = list()
for(i in seq(0.01,0.05,0.01)){
  A4[[length(A4)+1]] = bd.sim(20,20,0.15,i,0,0)
}
saveRDS(A4, "simdat4.rda")
```

B. DERIVATION OF THE ODE SOLUTION OF THE PURE BIRTH PROCESS

For the sake of clarity, we include here a derivation of the entries in Table 2 in the main text. Using the ODE

$$\frac{dI}{dt} = \alpha I$$

as a starting point, we can derive different observables. Thus, integrating the latter equation we can find the (total) number of infected

$$\frac{dI}{I} = \alpha dt \Rightarrow \log I(t) - \log I_0 = \alpha t \Rightarrow I(t) = e^{\alpha t}.$$

Hence, the number of new cases per unit time:

$$N(t) \equiv \frac{dI}{dt} = \alpha e^{\alpha t}$$

And the number of new cases in an interval of time Δt,

$$N_t \equiv \int_t^{t+\Delta t} N(t)dt = I(t+\Delta t) - I(t) = e^{\alpha t}\left(e^{\alpha \Delta t} - 1\right)$$

Finally, the total number of cases, in the absence of death, is the same as the total number that get infected, $I(t)$.

C. DERIVATION OF THE EQUATION FOR R^2

Equation (1) in the main text allows to calculate the probability of having a total number of infected cases at time t, $I(t)$, given the values of μ_A and σ_A.

$$P(I|\mu_A, \sigma_A, t) = \frac{\left(1 - e^{-\alpha t}\right)^{I-1} e^{-\frac{(\alpha-\mu_A)^2}{2\sigma_A^2} - \alpha t}}{\sqrt{2\pi\sigma_A^2}}, \quad I = 1, 2, \ldots$$

The moments of this probability distribution are given by

$$m_k = \int_{-\infty}^{\infty} d\alpha \sum_{I=1}^{\infty} I^k P(I|\mu_A, \sigma_A, t).$$

From this formula, we can calculate the moments explicitly, and, in particular, the mean and variance will be:

$$\langle I \rangle \equiv m_1 = e^{\mu_A t + \frac{\sigma_A^2 t^2}{2}} \tag{1}$$

and

$$\sigma_I^2 \equiv m_2 - m_1^2 = -e^{\frac{\sigma_A^2 t^2}{2} + \mu_A t} + 2e^{2t\left(\mu_A + \sigma_A^2 t\right)} - e^{t\left(2\mu_A + \sigma_A^2 t\right)} \tag{2}$$

It is worth noting that both the mean and the variance of I depend on μ_A and σ_A.

When $\mu_A \to \alpha$ and $\sigma_A \to 0$, and there is no parametric variability (*i.e.* all the variability is due to ”noise”), the expressions in Eqs. 2 and 3 of the main text are the same as the final two rows in Table 2 of the main text. In particular,

$$\sigma^2_{\text{noise}} = e^{\alpha t}\left(e^{\alpha t} - 1\right) \tag{3}$$

When σ_A is small, but not zero, as is the case in some of our simulations ($\sigma_A = 0.02$), we can use Taylor expansion of the terms in $e^{\sigma_A^2 t^2}$ to approximate expression (2), for times $t \ll 1/\sigma_A \simeq 50$, by

$$\sigma_I^2 = e^{\alpha t}\left(e^{\alpha t} - 1\right) + \frac{1}{2}\sigma_A^2 t^2 e^{\alpha t}\left(6e^{\alpha t} - 1\right) = \sigma^2_{\text{noise}} + \sigma^2_{\text{param}}$$

$$\sigma^2_{\text{param}} \equiv \sigma_A^2 \frac{1}{2} t^2 e^{\alpha t}\left(6e^{\alpha t} - 1\right).$$

We can simplify the latter expression further, for times $t \gg 1/\alpha$ (*i.e.*, when $e^{\alpha t} \gg 1$ and $e^{\alpha t} - 1 \approx e^{\alpha t}$), to obtain

$$\sigma_I^2 \simeq e^{2\alpha t} + 3\sigma_A^2 t^2 e^{2\alpha t} = \sigma_{\text{noise}}^2 (1 + 3\sigma_A^2 t^2)$$

Finally, we can define the analogous of the coefficient of determination. In the original least squares regression,

$$R^2 = \frac{\sigma_{\hat{y}}^2}{\sigma_{\hat{y}}^2 + \sigma_{\hat{r}}^2}$$

where $\hat{y}$ is the vector of predicted values (namely, $\hat{y} = ax + b$, where a and b are the fitted coefficients) and $r = y - \hat{y}$ the vector of residuals of the fit. In that regard, we define:

$$R^2 = \frac{\sigma_{\text{param}}^2}{\sigma_{\text{param}}^2 + \sigma_{\text{noise}}^2},$$

which gives a relative quantification of the parametric variance compared to the total variance of the stochastic process. For the pure birth case, we arrive at the following equation:

$$R^2 = \frac{\frac{1}{2}\sigma_A^2 t^2 e^{\alpha t} \left(6e^{\alpha t} - 1\right)}{e^{\alpha t} \left(e^{\alpha t} - 1\right) + \frac{1}{2}\sigma_A^2 t^2 e^{\alpha t} \left(6e^{\alpha t} - 1\right)} \simeq \frac{3\sigma_A^2 t^2}{1 + 3\sigma_A^2 t^2}$$

D. TABLES OF FITS TO THE SIMULATED DATA

Table I. Summary of parameters used in Experiment 1(columns $\mu_{A,B}$ and $\sigma_{A,B}$) and corresponding estimates (indicated with a hat, *e.g.*, $\hat{\mu}_A$). Confidence intervals are obtained using the likelihood curve method described in Sec. 2.2 of the main text.

Model	μ_A	$\hat{\mu}_A$	CI $\hat{\mu}_A$	σ_A	$\hat{\sigma}_A$	CI $\hat{\sigma}_A$	μ_B	$\hat{\mu}_B$	CI $\hat{\mu}_B$	σ_B	$\hat{\sigma}_B$	CI $\hat{\sigma}_B$
Det.	0.15	0.15	(0.15,0.16)	0	-	-	0	-	-	0	-	-
Det.	0.15	0.13	(0.1,0.17)	0	0.06	(0.05,0.11)	0	-	-	0	-	-
Det.	0.15	0.13	(0.13,0.14)	0.02	-	-	0	-	-	0	-	-
Det.	0.15	0.12	(0.1,0.15)	0.02	0.04	(0.03,0.06)	0	-	-	0	-	-
Det.	0.25	0.18	(0.17,0.18)	0	-	-	0.1	-	-	0	-	-
Det.	0.25	0.17	(0.12,0.21)	0	0.18	(0.13,0.21)	0.1	-	-	0	-	-
Det.	0.25	0.18	(0.17,0.18)	0.02	-	-	0.1	-	-	0.01	-	-
Det.	0.25	0.16	(0.12,0.21)	0.02	0.18	(0.13,0.21)	0.1	-	-	0.01	-	-
Det.	0.15	0.18	(0.17,0.2)	0	-	-	0	0.01	(0,0.07)	0	-	-
Det.	0.15	0.18	(0.13,0.22)	0	0	(0,0.04)	0		(0,Inf)	0	0.5	(0.06,Inf)
Det.	0.15	0.14	(0.13,0.15)	0.02	-	-	0	0	(0,0.03)	0	-	-
Det.	0.15	0.14	(0.11,0.16)	0.02	0.06	(0.03,0.09)	0		(0,Inf)	0		(0,Inf)
Det.	0.25	0.3	(0.27,0.33)	0	-	-	0.1	0.1	(0.04,0.16)	0	-	-
Det.	0.25	0.32	(0.26,0.42)	0	0.01	(0.01,0.07)	0.1	0.21	(0.1,0.4)	0	0.28	(0.2,0.64)
Det.	0.25	0.33	(0.3,0.36)	0.02	-	-	0.1	0.08	(0.01,0.14)	0.01	-	-
Det.	0.25	0.27	(0.2,0.38)	0.02	0	(0,0.17)	0.1	0.17	(0.09,0.33)	0.01	0.26	(0.14,0.39)
Sto.	0.15	0.15	(0.13,0.17)	0	-	-	0	-	-	0	-	-
Sto.	0.15	0.15	(0.15,0.18)	0	0.05	(0.03,0.11)	0	-	-	0	-	-
Sto.	0.15	0.13	(0.12,0.15)	0.02	-	-	0	-	-	0	-	-
Sto.	0.15	0.13	(0.1,0.16)	0.02	0.01	(0,0.05)	0	-	-	0	-	-
Sto.	0.25	0.17	(0.15,0.19)	0	-	-	0.1	-	-	0	-	-
Sto.	0.25	0.17	(0.13,0.2)	0	0.03	(0,0.15)	0.1	-	-	0	-	-
Sto.	0.25	0.17	(0.15,0.19)	0.02	-	-	0.1	-	-	0.01	-	-
Sto.	0.25	0.16	(0.12,0.2)	0.02	0.12	(0.05,0.2)	0.1	-	-	0.01	-	-
Sto.	0.15	0.17	(0.14,0.2)	0	-	-	0	0.02	(0,0.05)	0	-	-
Sto.	0.15	0.15	(0.12,0.19)	0	0.01	(0.01,0.06)	0	0.55	(0.05,2)	0		(0.03,Inf)
Sto.	0.15	0.14	(0.12,0.15)	0.02	-	-	0	0	(0,0.02)	0	-	-
Sto.	0.15	0.14	(0.11,0.16)	0.02	0	(0,0.03)	0		(0,Inf)	0		(0,Inf)
Sto.	0.25	0.27	(0.22,0.31)	0	-	-	0.1	0.1	(0.07,0.12)	0	-	-
Sto.	0.25	0.26	(0.21,0.29)	0	0	(0,0.03)	0.1	0.1	(0.07,0.13)	0	0	(0,0.19)
Sto.	0.25	0.25	(0.24,0.26)	0.02	-	-	0.1	0.1	(0.06,0.14)	0.01	-	-
Sto.	0.25	0.22	(0.17,0.27)	0.02	0	(0,0.05)	0.1	0.1	(0.05,0.4)	0.01	0	(0,0.4)

Table II. Summary of parameters used in Experiment 2 (columns μ_A and σ_A) and estimated (represented with the variables with a hat). Confidence intervals are obtained using the likelihood curve method described in Sec. 2.2 of the main text.

Model	O	μ_A	$\hat{\mu}_A$	CI $\hat{\mu}_A$	σ_A	$\hat{\sigma}_A$	CI $\hat{\sigma}_A$
Det.	10	0.150	0.143	(0.11,0.2)	0.020	0.090	(0.05,0.15)
Det.	20	0.150	0.159	(0.13,0.19)	0.020	0.038	(0.03,0.05)
Det.	30	0.150	0.151	(0.13,0.18)	0.020	0.037	(0.03,0.04)
Det.	40	0.150	0.155	(0.13,0.19)	0.020	0.047	(0.04,0.05)
Det.	50	0.150	0.161	(0.14,0.19)	0.020	0.036	(0.03,0.04)
Sto.	10	0.150	0.150	(0.11,0.21)	0.020	0.060	(0,0.15)
Sto.	20	0.150	0.168	(0.14,0.2)	0.020	0.002	(0,0.04)
Sto.	30	0.150	0.158	(0.13,0.19)	0.020	0.00001	(0,0.02)
Sto.	40	0.150	0.163	(0.14,0.2)	0.020	0.026	(0.01,0.05)
Sto.	50	0.150	0.153	(0.14,0.16)	0.020	0.017	(0.01,0.02)

Table III. Summary of parameters used in Experiment 3 (columns μ_A and σ_A) and estimated (represented with the variables with a hat). Confidence intervals are obtained using the likelihood curve method described in Sec. 2 of the main text.

Model	N	μ_A	$\hat{\mu}_A$	CI $\hat{\mu}_A$	σ_A	$\hat{\sigma}_A$	CI $\hat{\sigma}_A$
Det.	10	0.150	0.127	(0.09,0.18)	0.020	0.040	(0.03,0.09)
Det.	20	0.150	0.144	(0.12,0.19)	0.020	0.047	(0.04,0.08)
Det.	30	0.150	0.139	(0.11,0.16)	0.020	0.047	(0.04,0.07)
Det.	40	0.150	0.142	(0.12,0.17)	0.020	0.047	(0.04,0.06)
Det.	50	0.150	0.139	(0.12,0.16)	0.020	0.047	(0.04,0.07)
Sto.	10	0.150	0.137	(0.1,0.2)	0.020	0.058	(0,0.15)
Sto.	20	0.150	0.175	(0.13,0.21)	0.020	0.060	(0,0.12)
Sto.	30	0.150	0.156	(0.13,0.19)	0.020	0.051	(0.01,0.11)
Sto.	40	0.150	0.163	(0.14,0.2)	0.020	0.054	(0.01,0.1)
Sto.	50	0.150	0.165	(0.14,0.2)	0.020	0.057	(0.02,0.09)

Table IV. Summary of parameters used in Experiment 4 (columns μ_A and σ_A) and estimated (represented with the variables with a hat). Confidence intervals are obtained using the likelihood curve method described in Sec. 2 of the main text.

Model	μ_A	$\hat{\mu}_A$	CI $\hat{\mu}_A$	σ_A	$\hat{\sigma}_A$	CI $\hat{\sigma}_A$
Det.	0.150	0.143	(0.11,0.19)	0.010	0.048	(0.04,0.09)
Det.	0.150	0.148	(0.12,0.19)	0.020	0.047	(0.04,0.08)
Det.	0.150	0.127	(0.1,0.16)	0.030	0.051	(0.04,0.08)
Det.	0.150	0.130	(0.1,0.18)	0.040	0.051	(0.04,0.08)
Det.	0.150	0.175	(0.13,0.21)	0.050	0.066	(0.06,0.1)
Sto.	0.150	0.173	(0.13,0.21)	0.010	0.056	(0,0.12)
Sto.	0.150	0.178	(0.14,0.21)	0.020	0.045	(0,0.11)
Sto.	0.150	0.139	(0.11,0.19)	0.030	0.048	(0,0.11)
Sto.	0.150	0.147	(0.11,0.2)	0.040	0.100	(0.05,0.16)
Sto.	0.150	0.192	(0.15,0.24)	0.050	0.110	(0.06,0.16)
